# Dental implant in a multiple myeloma patient undergoing bisphosphonate therapy: A case report

**DOI:** 10.1002/ccr3.2150

**Published:** 2019-04-16

**Authors:** Mojtaba Bayani, Ali Arash Anooshirvani, Mohammad Keivan, Elham Mohammad‐Rabei

**Affiliations:** ^1^ Department of Periodontics, School of Dentistry Arak University of Medical Sciences Arak Iran; ^2^ Department of Hematology and Oncology, School of Medicine Arak University of Medical Sciences Arak Iran; ^3^ Private Practice Arak Iran; ^4^ Department of Orthodontics, School of Dentistry Arak University of Medical Sciences Arak Iran

**Keywords:** bisphosphonate, dental implant, multiple myeloma, osseointegration

## Abstract

Dental implant placement in patients with multiple myeloma undergoing bisphosphonates therapy could be accomplished; however, it can turn into a successful treatment for edentulous area and functionally stabilized for many years. But a meticulous case selection, proper medical consultation with physician, minimally invasive surgery, and other cautions must be considered.

## 1 BACKGROUND

The use of osseointegrated dental implants for the rehabilitation of fully edentulous and partially edentulous jaws has shown high success rates in the long term. The success of osseointegration depends mainly on the state of the host bone bed (in terms of quality and quantity) and its healing capacity.^1^ Furthermore, systemic factors may influence the healing of the bone around dental implants. Nevertheless, despite a reduced success rate caused by unfavorable systemic conditions, they may not always be absolute contraindications for bone augmentation and dental implant placement. This successful dental implant treatment in multiple myeloma (MM) patient is one of the rare successful cases in oral implantology field.^2^ According to study conducted by Najeeb et al, the several factors including severe periodontitis, site with preexisting inflammation or type IV quality bone, and anatomic site of implant can be associated with dental implant failure. They suggested the clinical examination and patient data have crucial role in dental implant treatment and reduce the risk of implant failure.^3^ Also, based on Javed et al study, the osteogenic surface coating can be used on implants enhanced osseointegration events in patients with poor bone quality like patients undergoing bisphosphonates (BPs) therapy.^4^

Multiple myeloma is a hematological malignancy that develops in plasma cells of the bone marrow. This malignancy usually affects patients within the age of 40‐70 years and is more prevalent in men.^5^ Since plasma cells are found in the bone marrow, bones are mainly affected in MM, and in fact, bone involvement is the most common complication, which involves up to 90% of the cases.^6^ Although oral lesions rarely occur in MM, jaw lesions are more common manifestations of this disease (with a prevalence of 8%‐15%). Maxillofacial manifestations of MM into bony lytic lesions are not uncommon, and jaw involvement is seen in almost 30% of the cases which may affect both the maxilla and the mandible.^7^

The treatments for MM intend to eliminate cancerous cells, control pain and the growth of tumor mass. For early stage MM or asymptomatic myeloma, there is evidence of osteoporosis, which can be managed by periodic infusions of BPs. Symptomatic myeloma, however, needs treatment as well as supportive therapy like pain relieving and nutritional therapy. Disease therapy includes drug therapy (such as BPs), steroids (such as prednisone and dexamethasone), thalidomide, lenalidomide, and bortezomib to treat patients in early stages. Other treatments include chemotherapy, radiation therapy, surgery, stem cell, and bone marrow transplantation.^8^

Medication‐related osteonecrosis of the jaw (MRONJ) is an avascular osteonecrosis associated with prolonged bisphosphonate therapy.^9^ Various risk factors for the development of MRONJ have been identified, which include invasive dental procedures and poor oral hygiene.^10^ Medication administration issues including duration of treatment, number of infusions, and infusion hours may affect the risk of MRONJ.^11^

Some problems that may be encountered in dental implant treatment of MM patients and the major precautions that should be considered are mentioned in brief:

- The jaw bony lytic lesions in MM, most commonly seen in the mandible, can alter the bone healing process after implant insertion. Placement of dental implants in these lytic areas should be avoided as it may increase rates of failure due to lack of proper osseointegration.^12^, ^13^
- As altered blood cell count may lead to an increased chance of infection, excessive bleeding during and after surgery, meticulous blood cell examination must be carried out before dental implant surgery to avoid possible complications.^14^
- Some MM patients have subclinical changes in bone quality that are not revealed in conventional radiographs; therefore, other accurate techniques such as cone beam computed tomography (CBCT) and positron emission tomography (PET) scan may be helpful to assess bone quality and quantity in these patients before implant placement.^12^, ^15^
- Single‐tooth implant insertion in MM patients is considered to have a better prognosis in contrast to full mouth implant insertion, and we should consider minimally invasive surgery protocols in these patients.^16^
- Before dental surgery, premedication with common antibiotics is recommended to reduce postoperation infection and improve bone healing consequences.^17^
- Flap design must involve small areas as larger incisions may increase postoperation complications such as swelling, hematoma, infection, and excessive bleeding.^13^
- Atraumatic tooth extraction when performing immediate implantation, conservative implant site preparation using sharp drills, and adequate irrigation can improve the bone healing process.^18^
- Primary stability of the fixture holds major importance in proper osseointegration.^19^
- As poor oral hygiene is considered a risk factor for MRONJ, maintaining good oral hygiene before and after surgery becomes of utmost importance.^20^

In this case, we reported the successful dental implant treatment in a MM patient is one of the rare successful cases in the field of oral implantology.

## 2 CASE PRESENTATION

**Figure 1:**
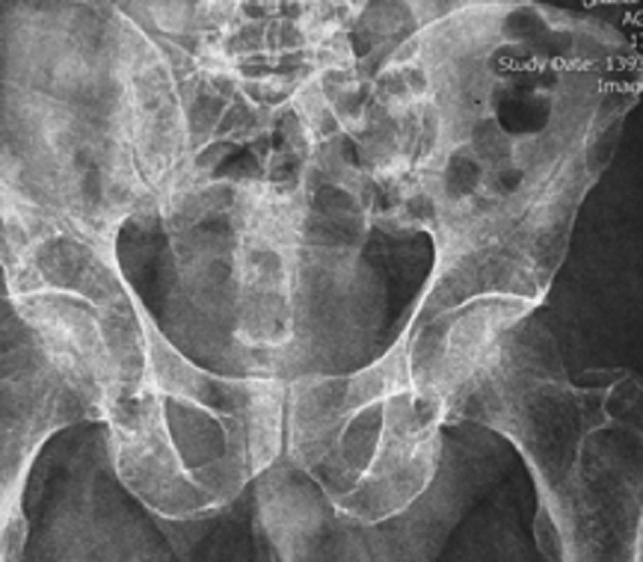
Lytic lesions in patient's hip radiography

**Figure 2:**
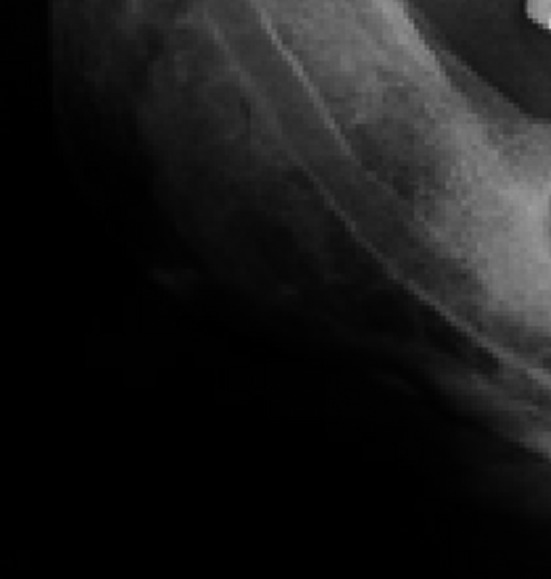
Lytic lesions could be seen in right aspect of mandibule

A 54‐year‐old man came to the private dental clinic with complaint of difficulty in mastication and esthetical concern for his upper anterior teeth. He was a nonsmoker and was diagnosed with IgG‐kappa type MM in November 2011. In the physical examination, he was diagnosed with MM. Bony metastasis was present at the time of diagnosis of the disease. A full radiographic skeletal survey showed multiple bony lesions at the ribs, femurs, and hip (Figures 1 and 2).

Panoramic view revealed bony lytic and punch out lesions at the right side of the mandible. This patient had no history of surgery. His weight had decreased by 7 kg, following 22 months of acute intravenous injection (IV) BP treatment after the last chemotherapy treatment session. His blood pressure was 130/80, and he had a normal breathing and pulse rate. Preoperative examination of his oral mucosa revealed no evidence of pathological lesions, and overall oral hygiene was good. The patient was felt healthy and was well nourished, alert, and cooperative. After thorough clinical examination, maxillary right first premolar was found missing.

After meticulous consulting sessions with the patient and discussing the advantages and disadvantages of all treatment options, he accepted to receive dental implant.

According to the patient's physician, the appropriate time for the surgery relied upon the patient's regular blood cell counts. This patient did not undergo any radiotherapy phases in the entire duration of his active IV BP treatment. He underwent chemotherapy for two separate sessions. After the last session of chemotherapy, the patient received monthly infusion of 3.5 mg of the IV BP drug zoledronate (Zometa; Novartis Pharmaceuticals Corporation) for a period of 22 months (from May 2014 to March 2016). As per the physician's recommendation, C‐terminal cross‐linking telopeptide (CTX) examination was carried out 6 months after stopping IV BP therapy. The CTX above of more than 150 was considered to be safe, in that the CTX was 289 pg/mL.

**Figure 3:**
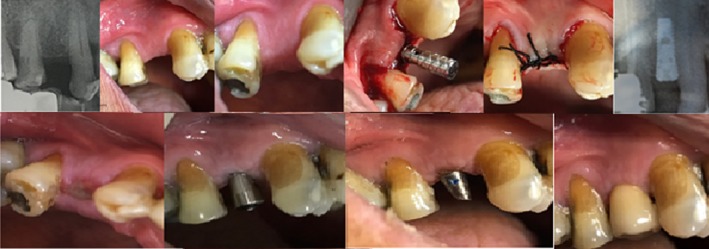
Clinical and radiographical procedure of dental implant placement

Before surgery, the patient was premeditated with 2 g of amoxicillin/clavulanic acid and 50 mg of diclofenac. A root form titanium dental implant (Superline; Dentium) of 3.6 mm in diameter and 10 mm in length was inserted under local anesthesia (Figure 3). The patient well‐tolerated the procedure and his vital signs were regularly monitored. Postoperative medications including antibiotics (1000 mg amoxicillin/clavulanic acid twice daily for 7 days, starting on the day of surgery), an analgesic (600 mg ibuprofen as required every 6 hours), and mouthwash (0.2% chlorhexidine twice daily for 2 weeks, starting on the day after surgery) were prescribed to the patient. Postoperative course and healing were unremarkable and typical. He was instructed to resume normal oral hygiene and chewing by week six. Postsurgical cleaning protocols, including oral hygiene instructions, were implemented at weeks 1, 2, 6, and 12.

**Figure 4:**
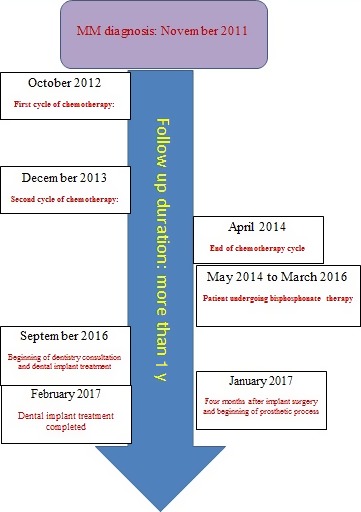
The time lines of patient's medical history and dental implant treatment

Four months after the implant insertion, the patient returned for punch removal of the gingiva overlying the implants. After 1 week, the appropriate impression copings were connected to the fixture. Polyether (Permadyne light and regular body; ESPE, Plymouth Meeting) was injected around the transfer copings and placed inside the custom tray using the dispenser. After laboratory procedure, abutment were positioned and torqued according to the manufacture's guidelines at 30 Ncm. After the surgical and prosthetic treatments were completed on February 2017, the patient was placed on a regular follow‐up for peri‐implant maintenance. The patient resumed IV BP therapy on May 2017. The oral hygiene regimen was implemented for this patient in a 6‐month recall. The last follow‐up (12 months after prosthetic delivery) showed minimum bone loss, as compared with the X‐rays taken immediately after the prosthetic delivery and the implant, and its restoration was successful. The patient was satisfied with the treatment (Figure 4).

## 3 DISCUSSION

The tooth replacement by dental implant has become a more attractive and efficient alternative approach, as compared to the conventional fixed and/or removable dental prosthetics.^21^

Some systemic conditions that affect the quality of jaw bones, such as osteoporosis, Paget disease, and MM, are also considered to be relative contraindications for dental implant placement. This must be taken into consideration in the dental implant treatment planning.^22^

We encountered three significant problems which could hinder the insertion of dental implant in safe condition: (a) MM seriously affects bone quality and the subsequent poor bone condition is not suitable for implant surgery, (b) MM affects immunological process and this patient was very susceptible to microbial infection, and (c) therapeutic medications of MM can alter soft and hard tissue biologically, thus affecting the osseointegration process in dental implant surgery and early failure of dental implant.

The BP family is the main medication used for the MM patients, which compromises the tissue healing capacity of these patients. Moreover, the intravenous BPs (eg, zoledronate) has destructive side effects, such as osteonecrosis of the jaw.^23^ A major dilemma that we encountered for the prevention of MRONJ was the exact duration of the treatment with intravenous BP for MM as there was no proven evidence from clinical trials. In the current case, the patient had a history of bisphosphonate medication for 22 months before dental implant. A study by Bagan et al^24^ showed a direct association between the use of intravenous bisphosphonate therapy and the development of MRONJ after dental implant placement, but this risk is lower from a simple tooth extraction in these patients. Some studies recommended patients taking IV BPs for more than 3 years with no local risk factors and those taking BPs for <3 years together with steroid therapy to take a 3‐to‐6‐month drug holiday prior to dental surgery,^25^ and in this patient, physician recommended a 6‐month drug holiday before dental implant placement.

Another important concern is considering an optimal surgical timing after chemotherapy. In fact, implant insertion must be done when the patient is healthy and at the best systemic condition after chemotherapy cycles. This is to ensure that there is complete osseointegration and a normal healing response of the manipulated tissue.

Some researchers advise the use of CTX test to evaluate the patient's risk of developing complications—such as MRONJ—following surgical interventions including dental implantation. Although there is no clear agreement between researchers about the effectiveness of CTX as a marker for MRONJ,^26^ in the current study a CTX examination was prescribed.

As Diz et al^27^ mentioned, there are few absolute contraindications for dental implant insertion, but some certain medical conditions may affect risk of failure. Until now, there has been little evidence found for the assumed contraindications.^15^ In the present case, the permission of the patient's physicians after consultation, normal blood cell counts, proper CTX value, proper bone quality assessed by preoperative imaging, proper tactile sense of surgeon during implant site preparation, good primary stability achievement, lack of any soft and hard tissue lesions is the surgical site or any periodontal pathology, no presence or history of any other systematics disease that it can alter osseointegration events such as diabetes, good oral hygiene, and adequate cooperation justified the planning of this treatment for the patient.

The implant therapy has been followed for 12 months, and the patient is currently healthy. Minimum amount of bone loss is seen around the implant. This study reveals that conservative and careful selection of MM patients with thorough medical consultation with the patient's physician/oncologist—in addition to optimal timing of the surgical and restoration phases of the implant treatment—led to a successful outcome to the dental implant treatment.

## 4 CONCLUSION

Although dental implant inserted for this patient showed excellent results after a 1‐year follow‐up, it would be of great benefit to determine the predictability of conservative implant treatments in patients with MM. Long‐term clinical trials on the placement of implants in patients with MM would be required to establish clear clinical guidelines. These guidelines would be based on well‐done clinical trial studies with exact and proper study designs for the management of adverse side effects derived from MM disease process and alteration in wound healing events due to medications such as intravenous bisphosphonate. Dental implant can be osseointegrated and remain functionally stable in patients with MM undergoing BP therapy.

## CONFLICT OF INTEREST

None declared.

## AUTHOR CONTRIBUTIONS

MB: performed dental implant insertion and its prosthodontics procedure, designed and implemented this presentation. AA: developed theoretical framework, monitored patient's health status and supervised this report. MK and EM‐R: contributed in writing and editing of this manuscript.

## ETHICAL APPROVAL

This case report was approved by the Ethical Practices Committee of Arak University of Medical Sciences, with reference number: IR ARAKMU.REC.1396.3002

## CONSENT FOR PUBLICATION

Written informed consent was obtained from the patient for publication of this case report and any accompanying images. A copy of the written consent is available for review by the Editor‐in‐Chief of this journal as an additional file."

## DATA ACCESSIBILITY

The datasets used and/or analyzed during the current report are available from the corresponding author [Dr Mojtaba Bayani] on reasonable request.
